# Curbing Inflammation

**DOI:** 10.1155/2013/468287

**Published:** 2013-10-01

**Authors:** R. Clive Landis, Christopher D. Buckley, Paulo Roberto B. Evora, David A. Hart

**Affiliations:** ^1^Edmund Cohen Laboratory for Vascular Research, The University of The West Indies, Bridgetown BB11115, Barbados; ^2^Rheumatology Research Group, School of Immunity & Infection, University of Birmingham, Birmingham B15 2TT, UK; ^3^Laboratory of Cardiovascular and Endothelial Function, Department of Surgery and Anatomy, Ribeirão Preto Faculty of Medicine, University of Sao Paulo, 900 Sao Paulo, SP, Brazil; ^4^McCaig Institute for Bone and Joint Health, University of Calgary, Calgary, AB, Canada T2N 4N1

## 1. Introduction

Inflammation stands at the centre of a range of natural and pathological processes, such as ageing, wound healing, infection, arthritis, autoimmune disease, cardiovascular disease, cancer, and the inflammatory response to surgery. It is well accepted that inflammation, in the right place and at the right time, is at the center of a healthy host response to natural or man-made stresses. However, systemic or runaway inflammation is pathological and it is incumbent to understand better how the inflammatory process is curbed, either naturally or with intervention. This special issue invited submissions to expand our understanding of contemporary mechanisms or approaches to curb the inflammatory response in three broad settings: (1) the systemic inflammatory response, (2) chronic inflammation, and (3) natural pathways in inflammatory resolution. The editors selected a good balance between original research papers and review articles that addressed each area of the call.

## 2. Curbing the Systemic Inflammatory Response

The systemic inflammatory response was first described in the critical care field for detecting and managing the whole body inflammatory reaction in acutely ill patients to sepsis, burns, or traumatic injuries [1]. It has also been adapted to recognize the iatrogenic triggering of a systemic inflammatory response in heart surgery due to contact activation of blood in the extracorporeal bypass circuit [2]. 

This special issue leads with three reviews discussing contemporary approaches towards curbing the systemic inflammatory response in sepsis, burn wounds, and neurogenic inflammation (A. M. Bernard and G. R. Bernard; J. A. Farina Jr. et al.; K. M. Lewis). Interleukin- (IL-) 6 and tumor necrosis factor (TNF-) *α* are identified in sepsis and burns in patients as important mediators at the top of the inflammatory cascade that may be targeted either through specific antibody therapy or through the early excision of full thickness burn tissue that may otherwise act as a cytokine reservoir. The papers echo the PIRO paradigm put forward to integrate how predisposing factors (P), the type of Insult (I) and the host response (R) combine to generate organ injury (O) [3]. This paradigm has helped shift therapeutic strategies towards attenuating the PIR steps before Organ injury (O) occurs. A key transition from PIR to O occurs at the level of endothelial barrier function that serves to protect organs from the inflammatory milieu in the circulation [4]. The loss of blood brain barrier (BBB) function is brought into focus by the paper on neurogenic inflammation, which discusses substance P as a key mediator of BBB permeability and, hence, as an attractive therapeutic target for attenuating injury to the central nervous system following traumatic brain injury, stroke, and meningitis. 

Preexisting factors have also been identified in heart surgery to explain the differential inflammatory response in patients towards a common iatrogenic insult [5]. An original paper in this special issue identified high white cell count at preadmission as a predictor of surgical complications, as assessed by frequency of 30-day readmission postsurgery (J. R. Brown et al.). White blood cell count is considered a simple but valid measure of the prevailing inflammatory status of a patient [6] and, hence, this research again fits the PIRO model of the systemic inflammatory response. 

## 3. Curbing Chronic Inflammation

This special issue attracted some thought-provoking reviews on areas of chronic inflammation that have been somewhat overlooked or less popular in the literature. One review pointed out the surprising decline in interest among researchers for investigating the role of inflammation *after* an acute myocardial infarction (AMI) as opposed to the causal role *prior* to an AMI [7] that is well covered in the literature (P. R. B. Evora et al.). This minority viewpoint, however, chimes with other advocates who have argued consistently that certain nagging gaps remain to be explained in understanding the role of inflammation on AMI outcomes [8, 9]. The review identifies endothelial injury as a gateway in the pathophysiology of ischemic heart disease and presents a conceptual overview for curbing this inflammatory disease process. Transient ischemia is a potential trigger for vascular permeability changes in endothelium and a submission on metabolic acidosis addresses this aspect as part of a strategy to curb inflammation (T. R. de Nadai et al.). Finally, an original paper compared the immunosuppressive properties of statins on T-cell immune responses with other conventional immunosuppressive agents, such as cyclosporine or dexamethasone (A. Jameel et al.). The paper demonstrated differential immunosuppressive properties of statins on T-cell proliferation, IL-1*β*, IL-17, and interferon- (IFN-) *γ* production depending on the type of immune activation.

## 4. Natural Pathways for Inflammatory Resolution

This special issue concludes with two research papers and a review addressing the topical issue of proresolving pathways [10] and how to harness these endogenous pathways to curb inflammation (B. J. Evans et al.; R. C. Landis et al.; T. J. Ahmed et al.). Two papers describe the evolution of the “wound healing” macrophage [11, 12], defined by the expression of CD163 (hemoglobin scavenging receptor), utilizing *in vitro* and *in vivo* approaches. CD163^+^ macrophages are shown to promote anti-inflammatory and cytoprotective pathways *in vitro* to limit prooxidant injury due to free heme, via pathways involving phosphatidylinositol-3-kinase activation, Akt phosphorylation, and IL-10 secretion. CD163^+^ cells were associated in the second paper with the phagocytic removal of apoptotic neutrophils, hence limiting potential histotoxic injury, with a shifting of the cytokine profile from proinflammatory mediators, including TNF-*α*, IL-6, IL-8/CXCL8, monocyte chemoattractant protein (MCP-1/CCL2), macrophage inflammatory protein (MIP1*α*/CCL3), MIP-1*β*/CCL4, and eotaxin (CCL11), towards immunoregulatory mediators, including macrophage-derived chemokine (MDC/CCL22), interferon-inducible protein (IP-10/CXCL10), and transforming growth factor (TGF)-*β*. Finally, an interesting review of proresolving mediators highlights the promise of melanocortin peptides as agents to limit the inflammatory process and protect tissues in a variety of preclinical models for inflammatory disease. 

## 5. Conclusion

The editors are pleased to present this special issue on curbing inflammation and trust it will be popular with a wide readership, from basic scientists, critical care physicians, surgeons, rheumatologists, cardiovascular researchers, and many more, since the inflammatory response impacts on so many fields and disease processes. The editors hope that this special issue will provide a conceptual framework and stimulate new ideas in the development of therapeutic strategies to curb the pathological inflammatory response.

*R. Clive Landis* *R. Clive Landis* *Christopher D. Buckley* *Christopher D. Buckley* *Paulo Roberto B. Evora* *Paulo Roberto B. Evora* *David A. Hart* *David A. Hart*
